# Free Vibration of Thin-Walled Composite Shell Structures Reinforced with Uniform and Linear Carbon Nanotubes: Effect of the Elastic Foundation and Nonlinearity

**DOI:** 10.3390/nano11082090

**Published:** 2021-08-17

**Authors:** Avey Mahmure, Francesco Tornabene, Rossana Dimitri, Nuri Kuruoglu

**Affiliations:** 1 Division of Mathematics, Graduate School of Natural and Applied Sciences, Usak University, Usak 64200, Turkey; mahmureavey@gmail.com; 2 Department of Innovation Engineering, University of Salento, 73100 Lecce, Italy; rossana.dimitri@unisalento.it; 3 Department of Civil Engineering of Faculty of Engineering and Architecture, Istanbul Gelisim University, Istanbul 34310, Turkey; nkuruoglu@gelisim.edu.tr

**Keywords:** CNT, elastic foundations, nonlinear free vibration, nonlinear frequency, shallow shell structures

## Abstract

In this work, we discuss the free vibration behavior of thin-walled composite shell structures reinforced with carbon nanotubes (CNTs) in a nonlinear setting and resting on a Winkler–Pasternak Foundation (WPF). The theoretical model and the differential equations associated with the problem account for different distributions of CNTs (with uniform or nonuniform linear patterns), together with the presence of an elastic foundation, and von-Karman type nonlinearities. The basic equations of the problem are solved by using the Galerkin and Grigolyuk methods, in order to determine the frequencies associated with linear and nonlinear free vibrations. The reliability of the proposed methodology is verified against further predictions from the literature. Then, we examine the model for the sensitivity of the vibration response to different input parameters, such as the mechanical properties of the soil, or the nonlinearities and distributions of the reinforcing CNT phase, as useful for design purposes and benchmark solutions for more complicated computational studies on the topic.

## 1. Introduction

The fast development of nanotechnology in recent years has encouraged the production of nanotubes, increasing their application in many engineering areas. The CNTs produced for the first time by Iijima in 1993, are increasingly used in various industries and commonly proposed as novel material due to their great potential [1,2]. One of the most important application areas of CNTs stems from their large use as reinforcement phase in traditional composites and polymers. The mechanical, thermal and electrical properties of composites reinforced with CNTs are significantly improved compared to more classical composites, along with an increased level of strength in their structural application [3,4,5]. For such reasons, CNTs are used in some areas of the defense industry, especially in rocket, aerospace and aviation industries, where high-precision computations are required [6,7,8]. Among various problems is the linear and nonlinear vibration behavior of composite shell structures involving the presence of different distributions of CNTs. Composite shells, indeed, can include uniform or nonuniform patterns of CNTs, depending on the desired mechanical properties of the structures [9,10,11,12,13,14,15,16,17,18,19,20,21,22,23,24]. In this framework, a pioneering work on the nonlinear vibrations of composite shell structures was represented by [9], which considered a linear distribution of CNTs within the material. Following this work, some linear and nonlinear free vibration problems were proposed in [10,11,12,13,14,15,16,17] and [18,19,20,21,22,23,24], respectively, for unconstrained shallow shells and panels reinforced by CNTs, while proposing different numerical methods to solve the related problems. 

The technological evolution of artificial materials and their manufacturing has expanded the application areas for such materials, improving the interest towards even more complicated and coupled problems, as well as the possible interactions of a structural member with its surrounding medium. Composite CNT-based shell structures resting on elastic foundations can be found in different civil and mechanical engineering applications, in nuclear power plants, etc. Among different possibilities to model an elastic foundation, the Pasternak and Winkler models are two common ways of describing its mechanical behavior, based on a different number of input parameters [25,26]. When modeling the vibrations of structural members containing CNTs, it is important to study the effect of the reinforcement phase and elastic foundations on the frequency–amplitude relationships. Up to date, most works from the literature have been devoted to the solution of linear vibration problems, by means of different numerical techniques [27,28,29,30,31,32,33,34,35]. More specifically, Tornabene et al. [27] examined the influence of Winkler–Pasternak foundations on the static and dynamic analysis of laminated double-curved shells and panels using the differential quadrature method. The same numerical approach was successfully proposed in [28] to study the vibration response of functionally graded carbon nanotube reinforced composite (FG-CNTRC) spherical shells on an elastic foundation. Zhang and Liew [29] applied an element-free approach to study the large deflection response of FG-CNTRC plates. Dinh and Nguyen [30] applied a fourth-order Runge–Kutta method and Galerkin method to solve the dynamic and vibration problem of FG-CNTRC truncated conical shells on elastic foundations. Shen and He [31] performed a large amplitude vibration analysis of FG-CNTRC double-curved panels on elastic foundation by applying a two-step perturbation approach, as also implemented in [32] to analyze the large amplitude vibration of FG shallow arches on a nonlinear elastic foundation. A further linear formulation was proposed by Sobhy and Zenkour [33] to study the vibrations of FG graphene platelet reinforced composite double-curved shallow shells on an elastic foundation; Sofiyev et al. [34,35] investigated the stability of CNTRC conical shells resting on an elastic foundation under hydrostatic pressure and combined loads in different settings.

Despite the considerable attention paid by the scientific literature to the linear vibration of shell structures, the nonlinear vibrations of CNT shallow shells resting on elastic foundations have not been adequately investigated. In this context, this paper aims to study the nonlinear free vibration behavior of thin-walled shell structures reinforced with CNTs and resting on an elastic Winkler- or Pasternak-type foundation, while proposing a Grigolyuk method to handle the problem. The organization of the rest of the paper is as follows: Section 2 recalls the basic theoretical aspects for both the shell-foundation interaction and nonlinear structural problem. Section 3 illustrates the analytical methodology applied to solve the problem, whose numerical investigation is presented and discussed in Section 4, while Section 5 closes the work with main comments and remarks. 

## 2. Theoretical Formulation

### 2.1. Description of Shell-Foundation Interaction Model

**Figure 1:**
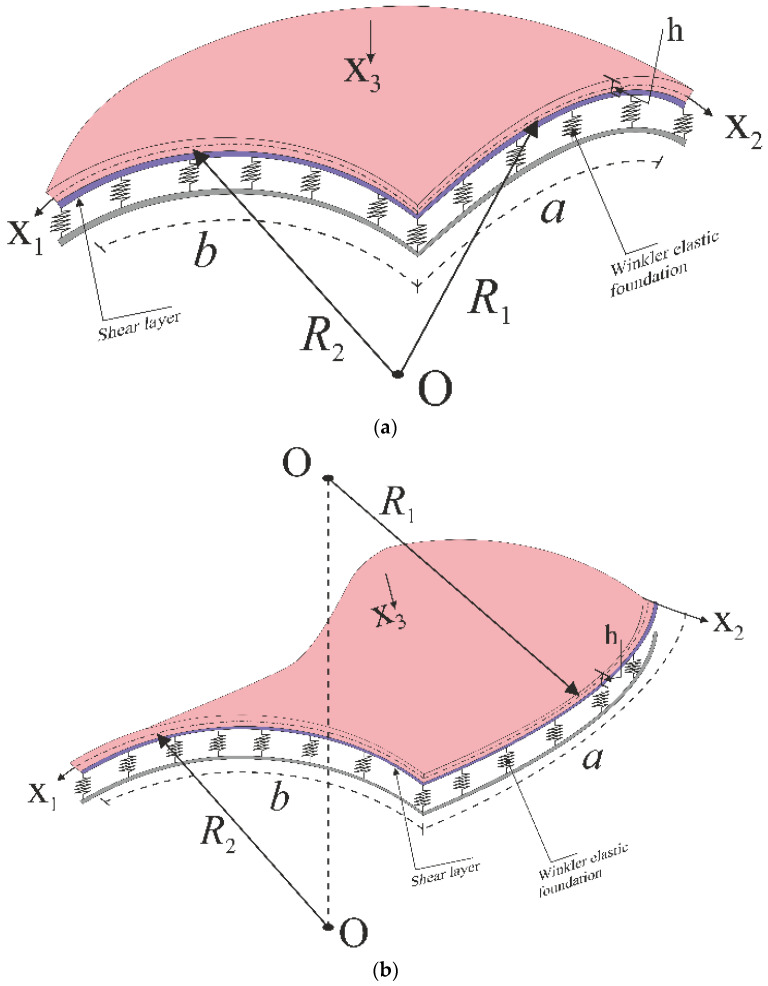
(**a**) Spherical and (**b**) hypar shallow shells reinforced with CNTs, resting on elastic foundations.

**Figure 2:**
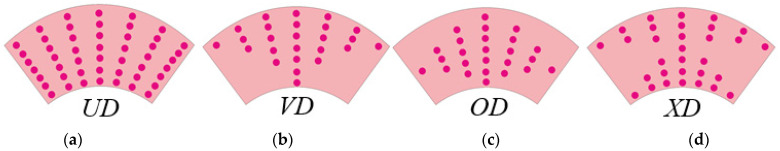
Cross-section of shallow shells with uniform and linearly patterned CNTs (**a**) UD, (**b**) VD, (**c**) OD and (**d**) XD.

Let us consider a composite spherical and hyperbolic paraboloidal (hypar) shallow shell reinforced with CNTs with length a, width b, thickness h and curvature radii $R_{1}$ and $R_{2}$, respectively (see Figure 1a,b). The Cartesian coordinate system $\left(x_{1},x_{2},x_{3}\right)$ is here assumed to define the shell geometry in its length, width and thickness direction, respectively. As also shown in Figure 1, both the spherical and hypar shallow shells are immersed in an elastic WPF, here modeled as follows [25,26]:

(1) $$K(w)=k_{w}w-k_{p}\left(\frac{\partial^{2}w}{\partial x_{1}^{2}}+\frac{\partial^{2}w}{\partial x_{2}^{2}}\right)$$

where $k_{w}$ (in Pa/m) is the Winkler spring stiffness and $k_{P}$ (in Pa⋅m) refers to the shear layer stiffness. When $k_{P}=0$, the foundation reverts to a Winkler-type elastic foundation (WF). The FG-CNTRC shell structures feature the following properties [9]

(2) $$\begin{array}{l} Y_{11}^{{\bar{x}}_{3}}=\eta_{1}V_{CN}^{{\bar{x}}_{3}}Y_{11}^{CN}+V_{m}E^{m},\;\;\;\frac{\eta_{2}}{Y_{22}^{{\bar{x}}_{3}}}=\frac{V_{CN}^{{\bar{x}}_{3}}}{Y_{22}^{CN}}+\frac{V_{m}}{E^{m}},\;\;\;\frac{\eta_{3}}{Y_{12}^{{\bar{x}}_{3}}}=\frac{V_{CN}^{{\bar{x}}_{3}}}{Y_{12}^{CN}}+\frac{V_{m}}{Y^{m}},\\ \nu_{12}=V_{CN}^{*}\nu_{12}^{CN}+V_{m}\nu^{{}_{m}},\;\;\;\rho_{1}^{{\bar{x}}_{3}}=V_{CN}^{*}\rho^{{}_{CN}}+V_{m}\rho^{{}_{m}},\;\;\;{\bar{x}}_{3}=x_{3}/h \end{array}$$

where the elastic properties for CNTs and matrix denoted as $Y_{ij}^{CN}(i,j=1,2)$, and $Y^{m}$,$G^{m}$, respectively; $\eta_{j}(j=1,2,3)$ refers to the efficiency parameters for CNTs; $V_{CN}^{{\bar{x}}_{3}}$ and $V^{m}$ stand for the volume fraction of CNTs and matrix, respectively, such that $V_{CN}^{{\bar{x}}_{3}}+V_{m}=1$. The density can be defined as

(3) $$V_{CN}^{*}=\frac{w_{CN}}{w_{CN}+(\rho^{{}_{CN}}/\rho^{{}_{m}})(1-w_{CN})}\;$$

whereas the volume fraction for shallow shells takes the following form (see Figure 2)

(4) $$V_{CN}^{{\bar{x}}_{3}}=\left\{ \begin{array}{l} UD\;\;\;\;\mathrm{at}\;\;\;\;\;\;\;V_{CN}^{*}\\ VD\;\;\;\;\mathrm{at}\;\;\;\;\left(1-{\bar{x}}_{3}\right)V_{CN}^{*}\\ OD\;\;\;\;\mathrm{at}\;\;\;\;\left(1+{\bar{x}}_{3}\right)V_{CN}^{*}\\ XD\;\;\;\;\mathrm{at}\;\;\;\;4\left|{\bar{x}}_{3}\right|\;V_{CN}^{*} \end{array}\right.$$

The strain field on the reference surface is governed by the following kinematic relations [36]

(5) $$\begin{array}{l} \varepsilon_{11}=\frac{\partial u}{\partial x_{1}}-\frac{w}{R_{1}}+\frac{1}{2}{\left(\frac{\partial w}{\partial x_{1}}\right)}^{2},\;\;\;\varepsilon_{22}=\frac{\partial v}{\partial x_{2}}-\frac{w}{R_{2}}+\frac{1}{2}{\left(\frac{\partial w}{\partial x_{2}}\right)}^{2}\\ \gamma_{12}=\frac{\partial v}{\partial x_{1}}+\frac{\partial u}{\partial x_{2}}+\frac{\partial w}{\partial x_{1}}\frac{\partial w}{\partial x_{2}} \end{array}$$

and the constitutive relations accounting for the von Karman nonlinearity within a classical shell framework are defined as [21]

(6) $$\left[\begin{array}{l} \tau_{11}\\ \tau_{22}\\ \tau_{12} \end{array}\right]=\left[\begin{array}{l} E_{11}^{{\bar{x}}_{3}}\;\;\;\;\;\;E_{12}^{{\bar{x}}_{3}}\;\;\;\;\;\;0\\ E_{21}^{{\bar{x}}_{3}}\;\;\;\;\;\;E_{22}^{{\bar{x}}_{3}}\;\;\;\;\;\;0\\ 0\;\;\;\;\;\;\;\;\;\;\;0\;\;\;\;\;\;E_{66}^{{\bar{x}}_{3}} \end{array}\right]\left[\begin{array}{l} e_{11}-{\bar{x}}_{3}\frac{\partial^{2}w}{\partial x_{1}^{2}}\\ e_{22}-{\bar{x}}_{3}\frac{\partial^{2}w}{\partial x_{2}^{2}}\\ \gamma_{12}-2{\bar{x}}_{3}\frac{\partial^{2}w}{\partial x_{1}\partial x_{2}} \end{array}\right]$$

with

(7) $$E_{ii}^{{\bar{x}}_{3}}=\frac{Y_{ii}^{{\bar{x}}_{3}}}{1-\nu_{ij}\nu_{ji}},\;\;\;E_{ij}^{{\bar{x}}_{3}}=\frac{\nu_{ji}Y_{ii}^{{\bar{x}}_{3}}}{1-\nu_{ij}\nu_{ji}}=E_{ji}^{{\bar{x}}_{3}},\;\;\;E_{66}^{{\bar{x}}_{3}}=Y_{ij}^{{\bar{x}}_{3}}(i,j=1,2)$$

### 2.2. Nonlinear Structural Model in the Presence of a PF

By using relations (1), (2), (5) and (6), the nonlinear governing equations for doubly curved shallow shells reinforced with a linear pattern of CNTs and resting on a WPF, attain the following form

(8) $$L_{11}(F)+L_{12}(w)+L_{13}(F,w)+K(w)=0$$

(9) $$L_{21}(F)+L_{22}(w)+L_{13}(w,w)=0$$

where F is a stress function and $L_{ij}(i=1,2,j=1,2,3)$ are differential operators defined as

(10) $$\begin{array}{l} L_{11}(F)=h\left[u_{12}\frac{\partial^{4}}{\partial x_{1}^{4}}+\left(u_{11}-2u_{31}+u_{22}\right)\frac{\partial^{4}}{\partial x_{1}^{2}\partial x_{2}^{2}}+u_{21}\frac{\partial^{4}}{\partial x_{2}^{4}}+\left(\frac{1}{R_{2}}\frac{\partial^{2}}{\partial x_{1}^{2}}+\frac{1}{R_{1}}\frac{\partial^{2}}{\partial x_{2}^{2}}\right)\right],\\ L_{12}(w)=-u_{13}\frac{\partial^{4}}{\partial x_{1}^{4}}-\left(u_{14}+2u_{32}+u_{23}\right)\frac{\partial^{4}}{\partial x_{1}^{2}\partial x_{2}^{2}}-u_{24}\frac{\partial^{4}}{\partial x_{1}^{4}}-\rho_{1}\frac{\partial^{2}}{\partial t^{2}},\\ L_{13}(F,w)=h\left(\frac{\partial^{2}}{\partial x_{2}^{2}}\frac{\partial^{2}}{\partial x_{1}^{2}}-2\frac{\partial^{2}}{\partial x_{1}\partial x_{2}}\frac{\partial^{2}}{\partial x_{1}\partial x_{2}}+\frac{\partial^{2}}{\partial x_{1}^{2}}\frac{\partial^{2}}{\partial x_{2}^{2}}\right),\;\;K(w)=k_{w}-k_{p}\left(\frac{\partial^{2}}{\partial x_{1}^{2}}+\frac{\partial^{2}}{\partial x_{2}^{2}}\right)\\ L_{21}(F)=h\left[q_{11}\frac{\partial^{4}}{\partial x_{2}^{4}}+(q_{12}+q_{21}+q_{31})\frac{\partial^{4}}{\partial x_{1}^{2}\partial x_{2}^{2}}+q_{22}\frac{\partial^{4}}{\partial x_{1}^{4}}\right],\\ L_{22}(w)=-q_{23}\frac{\partial^{4}}{\partial x_{1}^{4}}-\left(q_{24}+q_{13}-q_{32}\right)\frac{\partial^{4}}{\partial x_{1}^{2}\partial x_{2}^{2}}-q_{14}\frac{\partial^{4}}{\partial x_{2}^{4}}+\left(\frac{1}{R_{2}}\frac{\partial^{2}}{\partial x_{1}^{2}}+\frac{1}{R_{1}}\frac{\partial^{2}}{\partial x_{2}^{2}}\right),\\ L_{23}(w,w)=-{\left(\frac{\partial^{2}}{\partial x_{1}\partial x_{2}}\right)}^{2}+\frac{\partial^{2}}{\partial x_{1}^{2}}\frac{\partial^{2}}{\partial x_{2}^{2}} \end{array}$$

being t the time variable, and $\rho_{1}=\int_{-h/2}^{h/2}\rho_{1}^{{\bar{x}}_{3}}d{\bar{x}}_{3}$. Moreover, $u_{ij}$ are defined as

(11) $$\begin{array}{l} u_{11}=A_{11}^{1}q_{11}+A_{12}^{1}q_{21},\;\;u_{12}=A_{11}^{1}q_{12}+A_{12}^{1}q_{11},\;\;u_{13}=A_{11}^{1}q_{13}+A_{12}^{1}q_{23}+A_{11}^{2},\\ u_{14}=A_{11}^{1}q_{14}+A_{12}^{1}q_{24}+A_{12}^{2},\;\;u_{21}=A_{21}^{1}q_{11}+A_{22}^{1}q_{21},\;\;u_{22}=A_{21}^{1}q_{12}+A_{22}^{1}q_{22},\\ u_{23}=A_{21}^{1}q_{13}+A_{22}^{1}q_{23}+A_{21}^{1},\;\;u_{24}=A_{21}^{1}q_{14}+A_{22}^{1}q_{24}+A_{22}^{1},\;\;u_{31}=A_{66}^{1}q_{35},\;\\ \;u_{32}=A_{66}^{1}q_{32}+2A_{66}^{2}. \end{array}$$

with

(12) $$\begin{array}{l} q_{11}=\frac{A_{22}^{0}}{\Pi },\;\;q_{12}=-\frac{A_{12}^{0}}{\Pi },\;\;q_{13}=\frac{A_{12}^{0}A_{21}^{1}-A_{11}^{1}A_{22}^{0}}{\Pi },\;\;q_{14}=\frac{A_{12}^{0}A_{22}^{1}-A_{12}^{1}A_{22}^{0}}{\Pi },\;\;q_{21}=-\frac{A_{21}^{0}}{\Pi },\;\;q_{22}=\frac{A_{11}^{0}}{\Pi },\\ q_{23}=\frac{A_{11}^{1}A_{21}^{0}-A_{21}^{1}A_{11}^{0}}{\Pi },\;\;q_{24}=\frac{A_{12}^{1}A_{21}^{0}-A_{22}^{1}A_{11}^{0}}{\Pi },\;\;q_{31}=\frac{1}{A_{66}^{0}},\;\;q_{32}=-\frac{2A_{66}^{1}}{A_{66}^{0}},\;\;\Pi =A_{11}^{0}A_{22}^{0}-A_{12}^{0}A_{21}^{0},\\ A_{ij}^{i_{1}}=\int_{-h/2}^{h/2}E_{kk}^{{\bar{x}}_{3}}{\bar{x}}_{3}{}^{i_{1}}d{\bar{x}}_{3},(k=1,2,6;\;\;i_{1}=0,1,2),\;\;\;\;A_{ij}^{i_{1}}=\int_{-h/2}^{h/2}\nu_{ji}E_{ii}^{{\bar{x}}_{3}}{\bar{x}}_{3}{}^{i_{1}}d{\bar{x}}_{3},(i,j=1,2). \end{array}$$

## 3. Solution Procedure

In what follows, we provide an analytical solution to the problem of a simply-supported doubly-curved shell. Thus, the structural deflection can be approximated as [21,36]

(13) $$w=\bar{w}(t)\;\sin(\alpha_{1}x_{1})\;\sin(\alpha_{2}x_{2})$$

where $\bar{w}(t)$ is a function of time, $\alpha_{1}=\frac{m\pi }{a}$, $\alpha_{2}=\frac{n\pi }{b}$, in which m and n are the wave numbers in directions $x_{1}$ and $x_{2}$, respectively. By substitution of Equation (13) into Equation (9), we get the following expression for the stress function F

(14) $$F=\bar{w}(t)\left[c_{1}\bar{w}(t)\cos(2\alpha_{1}x_{1})+c_{2}\bar{w}(t)\cos(2\alpha_{2}x_{2})+c_{3}\sin(\alpha_{1}x_{1})\sin(\alpha_{2}x_{2})\right]$$

where $c_{j}(j=1,2,3)$ are defined as

(15) $$c_{1}=\frac{\alpha_{2}^{2}}{32\alpha_{1}^{2}q_{22}h},\;\;\;c_{2}=\frac{\alpha_{1}^{2}}{32\alpha_{2}^{2}q_{11}h},\;\;\;c_{3}=\frac{q_{23}\alpha_{1}^{4}+(q_{24}+q_{13}-q_{32})\alpha_{1}^{2}\alpha_{2}^{2}+q_{14}\alpha_{2}^{4}+\alpha_{1}^{2}/R_{2}+\alpha_{2}^{2}/R_{1}}{h\left[q_{11}\alpha_{2}^{3}+(q_{12}+q_{21}+q_{31})\alpha_{1}^{2}\alpha_{2}^{2}+q_{22}\alpha_{1}^{4}\right]}$$

By substituting Equations (13) and (15) into Equation (8) and by applying the Galerkin procedure in the domain $0\leq x_{1}\leq a$ and $0\leq x_{2}\leq b$, we obtain

(16) $$L(t)\equiv \frac{d^{2}\tilde{w}(t)}{dt^{2}}+\left(\omega_{wp}^{L}\right)\tilde{w}(t)+\theta_{1}{\tilde{w}}^{2}(t)+\theta_{2}{\tilde{w}}^{3}(t)=0$$

where $\tilde{w}(t)=\bar{w}(t)/h$, the quantities $\theta_{1},\;\;\theta_{2}$ are defined as

(17) $$\begin{array}{l} \theta_{1}=\frac{64h^{2}}{3ab}\frac{1}{\rho_{1}}\left(\frac{u_{12}\alpha_{1}^{4}c_{1}+u_{21}\alpha_{2}^{4}c_{2}}{\alpha_{2}\alpha_{1}}-\frac{\alpha_{1}\alpha_{2}c_{3}}{8}-\frac{c_{1}}{4R_{2}}\frac{\alpha_{1}}{\alpha_{2}}-\frac{c_{2}}{4R_{1}}\frac{\alpha_{2}}{\alpha_{1}}\right)\left[1-{\left(-1\right)}^{m}-{\left(-1\right)}^{n}+{\left(-1\right)}^{m+n}\right]\\ \theta_{2}=\frac{2h^{3}\alpha_{1}^{2}\alpha_{2}^{2}\left(c_{1}+c_{2}\right)}{\rho_{1}} \end{array}$$

and $\omega_{wp}^{L}$ is the frequency associated to the shallow structure resting on the PF at small deflections, defined as

(18) $$\begin{array}{l} \omega_{wp}^{L}=\frac{1}{\sqrt{\rho_{1}}}\{\left[\frac{\alpha_{1}^{2}}{R_{2}}+\frac{\alpha_{2}^{2}}{R_{1}}-u_{12}\alpha_{1}^{4}-(u_{11}-2u_{31}+u_{22})\alpha_{1}^{2}\alpha_{2}^{2}-u_{21}\alpha_{2}^{4}\right]hs_{3}\\ {\begin{array}{l} +u_{13}\alpha_{1}^{4}+(u_{14}+2u_{32}+u_{24})\alpha_{1}^{2}\alpha_{2}^{2}+u_{24}\alpha_{2}^{4}+k_{w}+k_{p}(\alpha_{1}^{2}+\alpha_{2}^{2}) \end{array}\}}^{1/2} \end{array}$$

The approximate solution of Equation (16) reads as follows

(19) $$\tilde{w}(t)=w_{0}\cos\left(\varpi_{NL}t\right)$$

where $w_{0}$ is the dimensionless amplitude, $\varpi_{NL}$ is the nonlinear frequency and the initial conditions are defined as

(20) $$\tilde{w}(t)|\begin{array}{l} t=0 \end{array}=w_{0}\;\;\;\mathrm{and}\;\;\;\frac{d\tilde{w}(t)}{dt}|\begin{array}{l} t=0 \end{array}=0$$

By combining the relations (16) and (19), we obtain an equation of the type L(t)=0. Thus, by applying the Grigolyuk method [37], one obtains

(21) $$\int_{0}^{\pi /2\varpi_{NL}}L(t)\cos\left(\varpi_{NL}t\right)dt=0$$

After integrating this last relation, we obtain the following nonlinear amplitude–frequency dependence

(22) $$\varpi_{wp}^{NL}=\sqrt{{\left(\omega_{wp}^{L}\right)}^{2}+\frac{8}{3\pi }\theta_{1}w_{0}+0.75\theta_{2}w_{0}^{2}}$$

and

(23) $$\varpi_{1wp}^{NL}=\varpi_{wp}^{NL}h\sqrt{\rho^{m}/Y^{m}}$$

The rational nonlinear-to-linear free vibration frequency (NLFVF / LFVF), $\varpi_{wp}^{NL}/\omega_{L},$ becomes

(24) $$\frac{\varpi_{wp}^{NL}}{\omega_{L}}=\sqrt{{\left(\frac{\omega_{wp}^{L}}{\omega_{L}}\right)}^{2}+\frac{8}{3\pi }\frac{\theta_{1}w_{0}}{\omega_{L}^{2}}+\frac{0.75\theta_{2}w_{0}{}^{2}}{\omega_{L}^{2}}}$$

where the linear-free vibration frequency $\omega_{L}$ for the unconstrained structure is defined as (18) and $k_{w}=k_{p}=0$ represents a special case. Based on Equations (22) and (24), we can treat different cases, namely shallow spherical shells (for $R_{1}=R_{2}$) or shallow hypar shells (for $R_{1}=-R_{2}$) resting on a PF, as well as shallow cylindrical panels ($R_{1}\to \infty$) or plates ($R_{1}\to \infty ,\;\;R_{2}\to \infty$), on a WPF.

The lowest values of $\varpi_{wp}^{NL}$, $\varpi_{1wp}^{NL}$ and $\frac{\varpi_{wp}^{NL}}{\omega_{L}}$ for shallow spherical and hypar shells on a PF are determined by minimizing Equations (20)–(22) depending on the vibration modes (*m, n*), for fixed values of the dimensionless amplitude $w_{0}$. 

## 4. Results and Discussion

The numerical investigation starts with a comparative evaluation of the dimensionless linear frequency parameters, $\omega_{1L}=\omega_{L}h\sqrt{\rho^{m}/Y^{m}}$, for isotropic shallow shells with respect to predictions from the literature [38] (see Table 1). 

Thus, we use the expression (18), while keeping $k_{w}=k_{p}=0,$ $Y_{11}^{m}=Y_{22}^{m}=Y^{m}=70\;\;\mathrm{Gpa},$ $\nu_{12}=\nu_{21}=\nu^{m}=0.3177,$$\rho^{m}=2.702\times 10^{3}\;\mathrm{kg}/m^{3}$ and the geometrical ratios a/b=1,   a/h=10. As visible from Table 1, our results match very well predictions from [38], for both spherical and hypar shell members; this proves the reliability and consistency of the proposed formulation. A further comparison with the literature [39,40] is also provided in terms of dimensionless linear frequencies for isotropic square plates resting on PFs with h/b=0.01,  a/b=1, $k_{w}=100D^{m}$ and $k_{p}=10D^{m}$. For this subcase, the relation (16) is computed for $R_{1}\to \infty ,\;\;R_{2}\to \infty$ and the dimensionless linear frequency parameter is determined as $\Omega^{Lwp}=\omega_{wp}^{L}{\left(b/\pi \right)}^{2}\sqrt{\rho^{m}h/D^{m}}$ in which $D^{m}=\frac{Y^{m}h^{3}}{12\left[1-{\left(\nu^{m}\right)}^{2}\right]}$, see [40]. Table 2 summarizes the results based on different approaches, with a consistent agreement between our formulation and findings from [39,40]. 

After this preliminary validation, we continue the analysis by computing the NLFVF of shallow spherical and hypar shells reinforced with a uniform and linear distribution of CNTs, and resting on a WF and PF. The selected shell members are made of polymethyl methacrylate (PMMA), as matrix, and single-walled CNTs, with geometrical properties $r_{1}=9.26\;\mathrm{nm},\;\;a_{1}=6.8\times 10^{-1}\;\mathrm{nm},$ $h_{1}=6.7\times 10^{-2}\;\mathrm{nm}$, as reinforcement. The mechanical properties for the CNT phase are $Y_{11}^{CN}=5646.6\;\mathrm{Gpa},$ $Y_{22}^{CN}=7080\;\;\mathrm{Gpa},$ $Y_{12}^{CN}=1944.5\;\mathrm{Gpa},$ $\;\nu_{12}^{CN}=0.175$, $\;\rho^{CN}=1.4\times 10^{3}\;\mathrm{kg}/m^{3}$; for PMMA, it is $Y^{m}=2.5\;\;\mathrm{Gpa},\;\;\nu^{m}=0.34,\;\;\rho^{m}=1.15\times 10^{3}\;\mathrm{kg}/m^{3}$. In line with [9], we also consider different efficiency parameters of CNT/matrix depending on the selected value of $V_{CN}^{*}$, as summarized in Table 3. As also listed in Table 4, we check for the variation of NLFVFs for both the selected shallow shells, while keeping different distributions of CNTs (i.e., UD, VD, OD and XD), and by varying the stiffness constants $\left(k_{w},\;k_{p}\right)$ for the elastic foundation under the three fixed values of $V_{CN}^{*}$ (0.12, 0.17, and 0.28). The frequency values are computed for $R_{1}=20h$, a=b, a=20h, (m,n)=(1,1) and $w_{0}=1.5$, with a clear increase in results for an increased value of the stiffness parameters, $k_{p}$ and/or $k_{w}$, for all the reinforcement assumptions. For fixed values of $k_{p}$, $k_{w}$, and $V_{CN}^{*},$ it also seems that OD and XD patterns of CNTs always provide the lowest and highest frequency values, respectively, independently of the selected shell geometry. 

The highest sensitivity of the response to the CNT dispersion within the matrix is observed for a fixed value of $V_{CN}^{*}=0.28$, with a maximum percentage variation with respect to a UD of 21.6%. At the same time, the largest foundation effect on $\varpi_{1wp}^{NL}$ occurs at $V_{CN}^{*}=0.12$ and OD patterns with a percentage variation of 51.5%. A PF also seems to affect the response more significantly compared to a W-EF, reaching the highest sensitivity with an OD-type reinforcement and $V_{CN}^{*}=0.12$, whereas the lowest sensitivity is obtained for a XD of CNTs with $V_{CN}^{*}=0.28$.

**Figure 3:**
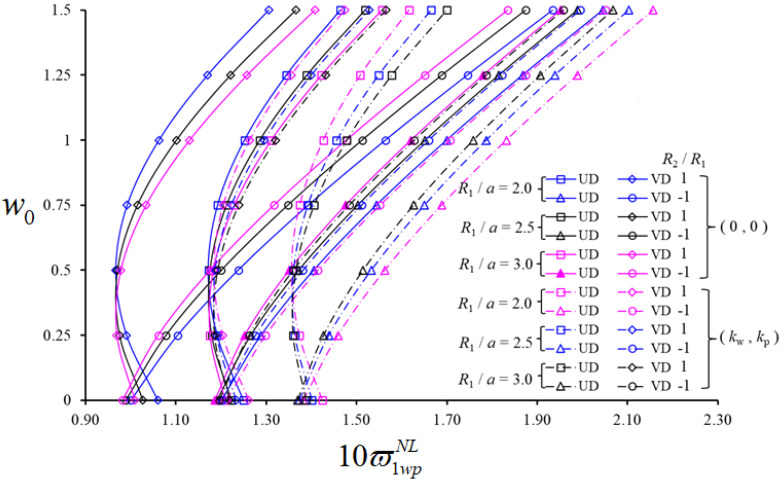
Variation in NLFVF for shallow spherical and hypar shells with UD- and VD-patterned CNTs on a PF versus $w_{0}$, with different geometrical ratios $R_{1}/a$.

As far as the sensitivity to the volume fraction is concerned, the largest influence is noticed for structures on a PF reinforced by XD CNTs with $V_{CN}^{*}=0.28$ and the lowest effect is obtained with an OD pattern and $V_{CN}^{*}=0.12$, respectively. In Figure 3, we plot the variation in NLFVFs for shallow spherical and hypar shells reinforced with a UD and VD of CNTs versus $w_{0}$, for a fixed value of $V_{CN}^{*}=0.28$, for three different geometrical ratios $R_{1}/a=2.0,2.5,3.0$, accounting (or not) for the presence of a surrounding WPF. The other parametric data are: a/b=0.5, a/h=15, (m,n)=(1,1), $k_{w}=4\times 10^{9}(N/m^{3})$ and $k_{p}=1.6\times 10^{4}(N/m)$. As visible in Figure 3, the NLFVF for hypar shells resting on a PF increases monotonically with $w_{0}$, whereas it varies non-monotonically for spherical shells on a PF with an initial decrease for $w_{0}\leq 0.5$, and a further increase for $w_{0}>0.5$. By comparing results among unconstrained spherical and hypar members, it seems that NLFVFs for hypar shells always reach higher values than spherical ones for all $w_{0}$; the NLFVFs for shallow spherical shells are usually higher for $w_{0}\leq 0.5$. 

The magnitude of NLFVFs for shell members with and without a PF can vary significantly under the same geometrical assumption $R_{1}/a$ and the same value of $w_{0}$. In addition, for an increasing rational value of $R_{1}/a$, the NLFVF values of spherical shells decrease for $w_{0}\leq 0.5$ and increase for $w_{0}>0.5$. The influence of CNT patterns on the NLFVF of hypar shells in the presence, or not, of a PF, decreases with a varying $w_{0}$. For spherical shells, instead, such an effect increases for $w_{0}\leq 0.5$ and decreases for $w_{0}>0.5$. Such sensitivity becomes more pronounced for $w_{0}>0.5$. More specifically, the influence of CNT patterns on the NLFVF for unconstrained hypar shells decreases from −16.71% to −5.41% due to the increase in $w_{0}$. For unconstrained spherical shells, the influence of CNT patterns increases from −15.20% to −17.51% in the range of $w_{0}\leq 0.5$, and decreases from −17.51% to −10.82% for $w_{0}>0.5$, under a fixed ratio $R_{1}/a=2$. The influence of different CNT patterns is more pronounced for unconstrained hypar shells, with the largest difference being approximately 1.00%; for spherical shells on a PF, the largest difference becomes approximately 1.8% due to an increased ratio $R_{1}/a$. 

The influence of CNT patterns on NLFVFs reduces with a maximum percentage of 4.76% and 4.61%, for spherical and hypar shells on PF, respectively. A pronounced effect of CNT patterns is also observed for both shallow shells in the presence, or not, of a PF, which is quantified as a percentage by 4.63% and 7.19%, respectively. The effect of a PF on NLFVF for both shells is approximately 6% greater for a VD pattern compared to a UD pattern. 

**Figure 4:**
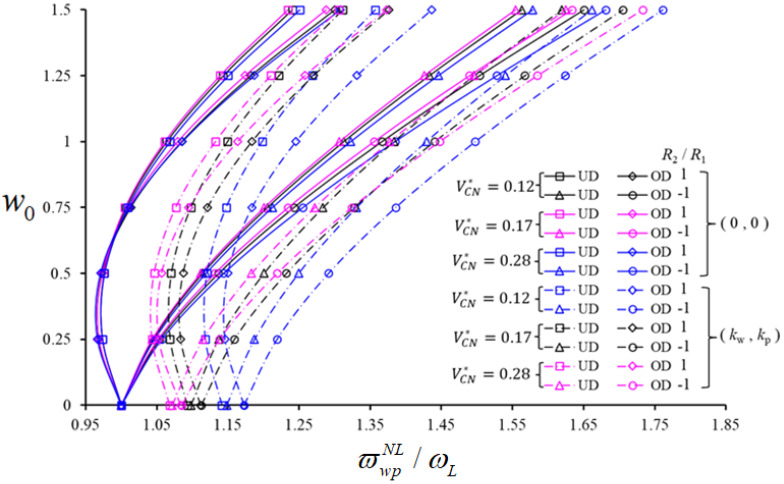
Variation in the NLFVF / LFVF ratio for shallow spherical and hypar shells with UD- and OD-patterned CNTs, in the presence/absence of a PF versus $w_{0}$, with different $V_{CN}^{*}$.

Variation in the NLFVF / LFVF ratio, for shallow spherical and hypar shells with UD and OD patterns versus $w_{0}$, is plotted in Figure 4, for $V_{CN}^{*}=0.12;\;\;0.17;\;\;0.28$, while keeping a/h=10 $R_{1}/a=2$, a/b=2, (m,n)=(1,1), $k_{w}=3\times 10^{9}(N/m^{3})$ and $k_{p}=1.5\times 10^{4}(N/m)$. As clearly visible in Figure 4, the NLFVF / LFVF ratio varies nonmonotonically for shallow spherical shells and monotonically for hypar shells, with an increased value of $w_{0}$ for all selected values of $V_{CN}^{*}$. 

Based on a comparison of the NLFVF / LFVF ratio for hypar shells with UD and OD patterns, in presence or absence of a PF, a higher variation is noticed for an OD pattern. More specifically, the NLFVF / LFVF ratio for spherical shells with an OD pattern becomes higher for all $w_{0}$ in presence of a PF, and for $w_{0}>0.5$ in the absence of a surrounding elastic medium. When the NLFVF / LFVF ratio is evaluated comparatively for spherical shells with different $V_{CN}^{*}$, the largest NLFVF / LFVF ratio for unconstrained spherical shells occurs at $V_{CN}^{*}=0.28$, and for spherical shells on PF at $V_{CN}^{*}=0.12$. For hypar shells in presence or not of a surrounding elastic medium, the highest NLFVF / LFVF ratio is always obtained for $V_{CN}^{*}=0.28$. It is also noticeable that this ratio becomes higher for hypar shells with and without the PF, as spherical and hypar shells are compared. The pattern effect on the NLFVF / LFVF ratio increases for unconstrained spherical shells (4%) and unconstrained hypar shells (5%), whereas the pattern effect on the $\varpi_{wp}^{NL}/\omega_{L}$ ratio in both shells on PF increases with $w_{0}$, accordingly. The most pronounced increase seems to be approximately equal to 2.5% for spherical shells, and approximately equal to 2.1% for hypar shells. In absence of a surrounding elastic medium, the influence of a CNT pattern on $\varpi_{wp}^{NL}/\omega_{L}$ for hypar shells becomes 1.9% more pronounced than unconstrained spherical shells on a PF.

The effect of a PF on $\varpi_{wp}^{NL}/\omega_{L}$ ratio decreases for an increased value of $w_{0}$, for both spherical and hypar shells, with a maximum percentage variation of 6% and 10%, respectively.

The variation in $\varpi_{wp}^{NL}/\omega_{L}$ with the PF is about 3.5% for spherical shells with an OD pattern of CNTs; this effect is 2.4% more pronounced for hypar shells. In addition, for a reinforcement phase with $V_{CN}^{*}=0.12$, the percentage variation of $\varpi_{wp}^{NL}/\omega_{L}$ for both shells under a PF is approximately 6% (or 9%) greater than that one for $V_{CN}^{*}=0.17$(or $V_{CN}^{*}=0.28$). 

**Figure 5:**
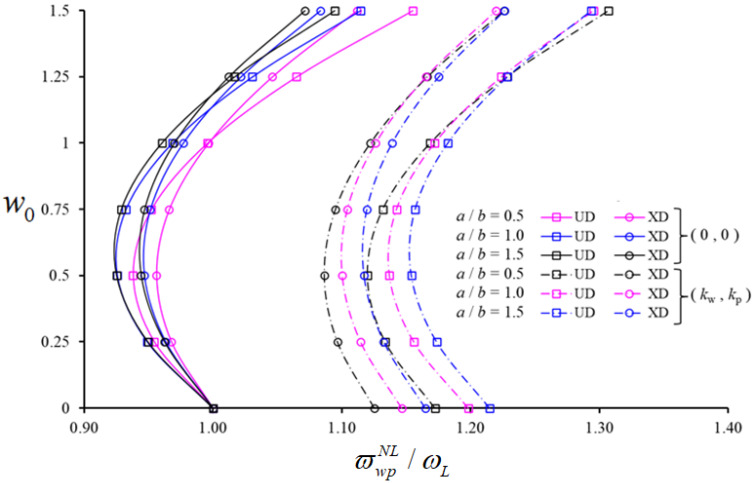
Variation in the NLFVF / LFVF ratio for shallow spherical shells containing UD- and XD-patterned CNTs in the presence/absence of a PF versus $w_{0}$, with different values of a/b.

Figure 5 shows the variation in the NLFVF / LFVF ratio of spherical shells on PF, reinforced with UD- and XD-patterned CNTs, against $w_{0}$, for $V_{CN}^{*}=0.17$. In this parametric study we also consider different values of a/b (i.e., a/b=0.5, 1.0, 1.5), along with $R_{1}/a=2$, a/h=15, (m,n)=(1,1), $k_{w}=3\times 10^{9}(\mathrm{Pa}/m)$ and $k_{p}=1.5\times 10^{5}(\mathrm{Pa}.m)$. As visible in Figure 5, for an increased value of a/b, the NLFVF / LFVF ratios of spherical shells with and without a PF vary nonmonotonically, with an increase after an initial decrease up to a minimum value. The NLFVF / LFVF ratio for UD patterns is larger than XD patterns for all $w_{0}$ (in presence of PF) and for $w_{0}>1$ (in absence of an elastic ground). The same ratio, for XD patterns, is larger than UD patterns, as $w_{0}\leq 1$ only in the absence of ground. 

For an increased value of a/b, the NLFVF / LFVF ratio of unconstrained spherical shells decreases for both patterns, while it decreases (or increases) when $w_{0}>1.25$ (or $w_{0}\leq 1.25$), for spherical shells on a PF. It is also observed that XD patterns effect on the NLFVF / LFVF ratio is higher in presence of a PF; it decreases/increases depending on the value of $w_{0}$, while it continuously decreases depending on the increase in a/b. Although the effect of PF on NLFVF / LFVF ratio is more pronounced for UD patterns, it decreases depending on the increase in $w_{0}$ and increases for an increased value of a/b. The minimum and maximum influence of PF on the NLFVF / LFVF ratio for spherical shells corresponds to a percentage variation of 10.24% and 24.71%, respectively.

**Figure 6:**
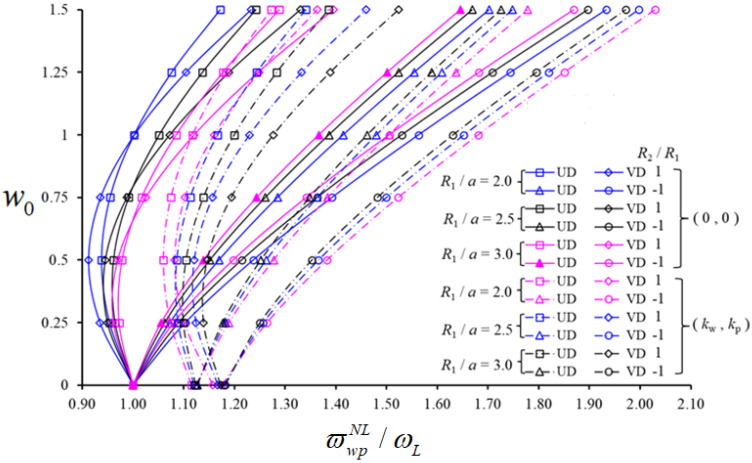
Variation in the NLFVF/LFVF ratio for shallow spherical and hypar shells with UD- and VD-patterned CNTs, in the presence/absence of a PF versus $w_{0}$ with different values of $R_{1}/a$.

Finally, in Figure 6 we plot the variation of NLFVF / LFVF ratio for UD- and VD-patterned spherical and hypar shells (in the presence or absence of a PF), versus $w_{0}$ for different $R_{1}/a$ ratios (i.e., $R_{1}/a=2.0,2.5,3.0$), while keeping $V_{CN}^{*}=0.28$, a/b=0.5, a/h=15, (m,n)=(1,1), $k_{w}=3\times 10^{9}(N/m^{3})$ and $k_{p}=1.5\times 10^{5}\;(N/m)$. The NLFVF / LFVF ratio of hypar shells on PF increases with $w_{0}$, while it varies nonmonotonically with $R_{1}/a$. Similarly, the NLFVF / LFVF ratio of spherical shells on PF decreases first and then increases for an increased value of $w_{0}$, while always increasing for an increased $R_{1}/a$ ratio. The NLFVF / LFVF ratio of hypar shells for VD patterns with and without a PF, as well as for spherical shells resting on a PF, is greater than the same shells reinforced uniformly by CNTs. The NLFVF / LFVF ratio of unconstrained spherical shells with VD patterns is higher than those with UD patterns, at least when $w_{0}\leq 0.75$; the contrary occurs for $w_{0}>0.75$. Based on a comparative evaluation of both geometries, the NLFVF / LFVF ratios of UD- and VD-reinforced spherical shells on a PF are lower than hypar shells. The influence of VD patterns on $\varpi_{wp}^{NL}/\omega_{L}$ for spherical shells is higher than hypar shells, with a maximum increase of 3.2% or 0.6%, respectively, depending on the increase in $R_{1}/a$. Looking at the influence of the foundation on $\varpi_{wp}^{NL}/\omega_{L}$ for spherical shells with a UD of CNTs, it decreases first, up to a minimum value, then increases for an increased value of $w_{0}$. A monotonic decrease is differently observed for hypar shells. Depending on the increase of $R_{1}/a$, the effect of PF is lower than 2% for spherical shells, and lower than 1.5% for hypar shells. 

## 5. Conclusions

In this work, the Donnell’s nonlinear shell theory is applied to study the free vibration behavior of composite shell structures reinforced by uniform and linearly patterned CNTs resting on a PF. Once the basic relations for composite shallow shells reinforced by CNTs on WPFs are established, the partial differential equations of nonlinear motion are derived, taking into account the von Karman nonlinearity. These equations are solved here by means of the Galerkin and Grigolyuk methods in terms of linear and nonlinear free vibrations for inhomogeneous nanocomposite construction members such as plates, panels, spherical and hyperbolic paraboloidal (hypar) shallow shells. The accuracy of the results in the current study has been confirmed by means of a successful comparison with reliable predictions from the literature. After this preliminary validation, a detailed numerical analysis is performed, including the effect of nonlinearity, CNT patterns and volume fraction on the nonlinear frequency response. Based on a large systematic investigation, the analytical results could serve as valid benchmark solutions for further computational studies on the topic, as well as for design purposes. Among the most useful insights, it is found that the variation rate of NLFVFs for both shallow shells with linearly patterned CNTs decreases, while remaining constant for different elastic foundations with an increased stiffness. For both shallow shells, a single- or dual-parameter elastic foundation yields an increase in NLFVFs, where the NLFVF decreases for VD, OD and XD patterns, as foundation coefficients increase. Moreover, the influence of PF on the $\varpi_{1wp}^{NL}$ for shallow spherical and hypar shells reinforced with CNTs has revealed as more pronounced than that of a WEF. The highest influence of PF on NLFVF values is observed with an OD pattern of CNTs for $V_{CN}^{*}=0.12$, whereas the smallest effect is observed with an XD pattern of CNTs for $V_{CN}^{*}=0.28$, respectively, when the influences of PF on NFVFs for spherical or hypar shells are compared to each other. Based on a comparative evaluation of the nonlinear vibration response for both shallow shells, the largest effect of the PF on NLFVFs is observed for a XD CNT-based reinforcement with a volume fraction, $V_{CN}^{*}=0.28$, whereas the smallest effect occurs for an OD pattern and $V_{CN}^{*}=0.12$. At the same time, the NLFVF of hypar shells on PF increases continuously for an increased $w_{0}$; it decreases when $w_{0}\leq 0.5$ for spherical shells on PF and increases for $w_{0}>0.5$. The pattern effect on $\varpi_{wp}^{NL}/\omega_{L}$ ratio for spherical shells (4%) and hypar shells (5%) increases with $w_{0}$ in absence of a PF. Based on the parametric study, the influence of the PF on $\varpi_{wp}^{NL}/\omega_{L}$ ratio seems to be more pronounced for a UD pattern, but it decreases depending on the increase in $w_{0}$ and increases for an increased geometrical ratio a/b. Moreover, the rational value of $\varpi_{wp}^{NL}/\omega_{L}$ for a UD pattern is higher than a XD pattern, for all $w_{0}$ in presence of an elastic medium, and for $w_{0}>1$ in absence of an elastic medium. The same ratio for an XD pattern is larger than the one for a UD pattern, as $w_{0}\leq 1$ only in absence of an elastic ground. Finally, for a VD pattern, the rational value of $\varpi_{wp}^{NL}/\omega_{L}$ for spherical shells is greater than the hypar shells, with a maximum increase of 3.2% in lieu of 0.6%, as found for hypar shells, depending on the increase in the geometrical ratio $R_{1}/a$. 

## Figures and Tables

**Table 1:** Comparative evaluation with the literature of $\omega_{1L}$ for different shallow structural members made of an isotropic material.

Structural Members	$\frac{a}{R_{1}}$	$\frac{b}{R_{2}}$	$\omega_{1L}=\omega_{L}h\sqrt{\rho^{m}/Y^{m}}$	
			Alijani [38]	Present Study
Spherical shell	0.5	0.5	0.0779	0.0781
Hypar shell	0.5	−0.5	0.0597	0.0600

**Table 2:** Comparison of dimensionless frequency parameters for square plates on a PF (h/b=0.01, a/b=1, $k_{w}=100D^{m},\;\;k_{p}=10D^{m}$ ).

Studies	Mode Number		
	$\Omega_{1,1}^{Lwp}$	$\Omega_{1,2}^{Lwp}$	$\Omega_{2,1}^{Lwp}$
Zhou et al. [39]	2.6551	5.5717	5.5717
Wang et al. [40]	2.6551	5.5717	5.5717
Present study	2.6557	5.5761	5.5761

**Table 3:** Typical properties of CNT/matrix efficiency parameters depending on the volume fraction of CNTs.

$V_{CN}^{*}$	$\eta_{1}$	$\eta_{2}$	$\eta_{3}$
0.12	0.137	1.022	0.715
0.17	0.142	1.626	1.138
0.28	0.141	1.585	1.109

**Table 4:** Variation in NLFVF for shallow spherical and hypar shells with CNTs resting on a W-EF and PF with various foundation elastic parameters, versus $V_{CN}^{*}$.

		$\varpi_{1wp}^{NL}\times 10$ $\left(R_{2}=R_{1}\right)$											
	$V_{CN}^{*}$	**0.12**				**0.17**				**0.28**			
$k_{p}$	$k_{w}$	UD	VD	OD	XD	UD	VD	OD	XD	UD	VD	OD	XD
0	0	0.445	0.388	0.362	0.540	0.569	0.506	0.476	0.680	0.615	0.517	0.493	0.782
0	$0.7\times 10^{9}$	0.503	0.452	0.431	0.589	0.614	0.557	0.530	0.719	0.656	0.566	0.544	0.815
	$1.0\times 10^{9}$	0.526	0.478	0.457	0.608	0.633	0.577	0.551	0.735	0.673	0.586	0.564	0.829
	$1.3\times 10^{9}$	0.548	0.502	0.482	0.627	0.651	0.597	0.572	0.750	0.690	0.605	0.584	0.843
$9\times 10^{4}$	$0.7\times 10^{9}$	0.583	0.540	0.522	0.658	0.681	0.629	0.605	0.776	0.717	0.636	0.66	0.685
	$1.0\times 10^{9}$	0.602	0.561	0.544	0.676	0.697	0.647	0.624	0.791	0.733	0.653	0.634	0.878
	$1.3\times 10^{9}$	0.622	0.581	0.565	0.693	0.714	0.665	0.642	0.805	0.748	0.670	0.652	0.891
$11\times 10^{4}$	$0.7\times 10^{9}$	0.599	0.557	0.540	0.672	0.694	0.644	0.621	0.788	0.730	0.650	0.631	0.876
	$1.0\times 10^{9}$	0.618	0.578	0.561	0.690	0.711	0.662	0.639	0.803	0.745	0.667	0.649	0.889
	$1.3\times 10^{9}$	0.637	0.598	0.581	0.706	0.727	0.679	0.657	0.817	0.760	0.684	0.666	0.901
$13\times 10^{4}$	$0.7\times 10^{9}$	0.615	0.574	0.557	0.687	0.708	0.659	0.636	0.800	0.743	0.664	0.646	0.886
	$1.0\times 10^{9}$	0.634	0.594	0.578	0.703	0.724	0.676	0.654	0.815	0.758	0.681	0.663	0.899
	$1.3\times 10^{9}$	0.652	0.614	0.598	0.720	0.740	0.693	0.671	0.829	0.773	0.698	0.680	0.912
		$\varpi_{1wp}^{NL}\times 10$ $\left(R_{2}=-R_{1}\right)$											
0	0	0.911	0.878	0.878	0.960	1.095	1.055	1.055	1.155	1.376	1.325	1.324	1.453
0	$0.7\times 10^{9}$	0.941	0.909	0.909	0.988	1.119	1.080	1.080	1.178	1.395	1.345	1.344	1.471
	$1.0\times 10^{9}$	0.953	0.921	0.922	1.000	1.129	1.091	1.091	1.188	1.403	1.353	1.352	1.479
	$1.3\times 10^{9}$	0.965	0.934	0.934	1.012	1.140	1.101	1.101	1.198	1.411	1.361	1.361	1.486
$9\times 10^{4}$	$0.7\times 10^{9}$	0.986	0.955	0.955	1.031	1.157	1.119	1.119	1.214	1.425	1.375	1.375	1.499
	$1.0\times 10^{9}$	0.997	0.967	0.967	1.043	1.167	1.129	1.129	1.223	1.433	1.384	1.383	1.507
	$1.3\times 10^{9}$	1.009	0.979	0.979	1.054	1.177	1.139	1.140	1.233	1.440	1.392	1.391	1.514
$11\times 10^{4}$	$0.7\times 10^{9}$	0.995	0.965	0.965	1.041	1.165	1.127	1.128	1.122	1.431	1.382	1.382	1.505
	$1.0\times 10^{9}$	1.007	0.977	0.977	1.052	1.175	1.138	1.138	1.231	1.439	1.390	1.390	1.513
	$1.3\times 10^{9}$	1.018	0.989	0.989	1.063	1.185	1.148	1.148	1.240	1.447	1.398	1.398	1.520
$13\times 10^{4}$	$0.7\times 10^{9}$	1.005	0.975	0.975	1.050	1.173	1.136	1.136	1.229	1.438	1.389	1.388	1.512
	$1.0\times 10^{9}$	1.016	0.987	0.987	1.061	1.183	1.146	1.146	1.239	1.445	1.397	1.396	1.519
	$1.3\times 10^{9}$	1.028	0.999	0.999	1.072	1.193	1.156	1.156	1.248	1.453	1.405	1.405	1.527
